# Neurological involvement in Kawasaki disease: a retrospective study

**DOI:** 10.1186/s12969-020-00452-7

**Published:** 2020-07-14

**Authors:** Xiaoliang Liu, Kaiyu Zhou, Yimin Hua, Mei Wu, Lei Liu, Shuran Shao, Chuan Wang

**Affiliations:** 1 Department of Pediatric Cardiology, West China Second University Hospital, Sichuan University, No. 20, 3rd section, South Renmin Road, Chengdu, 610041 Sichuan China; 2 Key Laboratory of Birth Defects and Related Diseases of Women and Children (Sichuan University), Ministry of Education, Chengdu, Sichuan China; 3 Key Laboratory of Development and Diseases of Women and Children of Sichuan Province, West China Second University Hospital, Sichuan University, Chengdu, Sichuan China; 4 The Cardiac Development and Early Intervention Unit, West China Institute of Women and Children’s Health, West China Second University Hospital, Sichuan University, Chengdu, Sichuan China; 5 West China Medical School of Sichuan University, Chengdu, Sichuan China

**Keywords:** Neurological involvement, Kawasaki disease, Intravenous immunoglobulin resistance, Coronary artery lesions, Vasculitis

## Abstract

**Background:**

Kawasaki disease (KD) is an acute, self-limiting systemic vasculitis that predominately affects children. Neurological involvement is a known complication of KD, however, its association with KD severity remains elusive. We aimed to systematically describe the general manifestations of neurological involvement in KD, determine whether neurological involvement is a marker of disease severity in patients with KD, and assess the relationship of such involvement with intravenous immunoglobulin (IVIG) resistance and coronary artery lesions (CALs).

**Methods:**

We retrospectively reviewed data from 1582 patients with KD between January 2013 and December 2017. Profiles of patients with neurological symptoms (group A, *n* = 80) were compared to those of gender- and admission date-matched patients without neurological involvement (group B, *n* = 512). Multivariate logistic regression analyses were performed to determine whether neurological involvement was significantly associated with IVIG resistance.

**Results:**

Neurological involvement was observed in 5.1% (80/1582) of patients with KD. The neurological manifestations were diffuse, presenting as headache (13/80, 16.3%), convulsions (14/80, 17.5%), somnolence (40/80, 50.1%), extreme irritability (21/80, 26.3%), signs of meningeal irritation (15/80, 18.8%), bulging fontanelles (7/80, 8.8%), and facial palsy (1/80, 1.3%). Neurological symptoms represented the initial and/or predominant manifestation in 47.5% (38/80) of patients with KD. The incidence of IVIG resistance and levels of inflammatory markers were higher in group A than in group B. However, neurological involvement was not an independent risk factor for IVIG resistance or CALs.

**Conclusion:**

Rates of neurological involvement were relatively low in patients with KD. Neurological involvement was associated with an increased risk of IVIG resistance and severe inflammatory burden. Our results highlight the need for pediatricians to recognize KD with neurological involvement and the importance of standard IVIG therapy.

**Trial registration:**

Retrospectively registered.

## Background

Kawasaki disease (KD) is an acute, self-limiting form of systemic vasculitis that predominately affects children between the ages of 6 months and 5 years. Clinically, KD is typically characterized by persistent fever, bilateral non-exudative conjunctivitis, erythema of the lips and oral mucosa, changes in the extremities, rash, and cervical lymphadenopathy [1]. In addition, KD can affect multiple organs and tissues including the pulmonary and gastrointestinal systems, thereby increasing the risk of coronary artery lesions (CALs) associated with delayed diagnosis and/or intravenous immunoglobulin (IVIG) resistance [2–4].

Although neurological involvement has been identified as a complication of KD, with an incidence of 1 to 30% [5, 6], its clinical features are diverse, and there are few reported cases in the literature [7–18]. Thus, pediatricians may be unfamiliar with the characteristics, course, clinical manifestations, diagnosis, treatment, and prognosis of KD with neurological involvement. Furthermore, the familiar diagnostic features of KD often appear sequentially and become obvious only after the initial neurological presentation, possibly resulting in unnecessary/inadequate treatment and/or therapeutic delay, and in turn increasing the risk of developing IVIG resistance and CALs [19]. The associations between neurological involvement and IVIG resistance and CALs are, however, not well clarified. Therefore, in the present study, we aimed to systematically describe the general manifestations of neurological involvement in KD, determine whether neurological involvement is a marker of disease severity in patients with KD, and assess the relationship of such involvement with IVIG resistance and CALs.

## Methods

We retrospectively reviewed data for a total of 1582 patients diagnosed with KD between January 2013 and December 2017 at the West China Second University Hospital of Sichuan University (WCSUH-SCU). Written informed consent was obtained from the parents following a full explanation of the nature of the study. The University Ethics Committee on Human Subjects at Sichuan University approved the study.

Neurological involvements were recorded, including somnolence, extreme irritability, headache, convulsions, signs of meningeal irritation, bulging fontanelles, hemiplegia, facial nerve palsy, sensorineural hearing loss, and aseptic meningitis (pleocytosis in the cerebrospinal fluid (CSF) ≥15 × 10^6^/L). The presences of somnolence and extreme irritability were documented in accordance with standard definitions of Richmond Agitation Sedation Scale (RASS) [20, 21] when reported by caregivers and/or directly observed during the acute stage of hospitalization. Headache was defined based on clinical history and physical examination using an age-appropriate pain assessment scale (i.e., the Face, Legs, Activity, Cry, Consolability Scale (FLACC Scale) [22], Wong-Baker Faces Pain Rating Scale [23]). Abnormal CSF results were defined as follows: pleocytosis ≥15 × 10^6^/L, protein level > 430 mg/L, glucose < 2.8 mmol/L, chloride < 120 mmol/L. The convulsions, signs of meningeal irritation, bulging fontanelles, aseptic meningitis, and facial nerve palsy, were identified by clinical history, CSF results, physical and neuroimaging examinations. Congenital, trauma-related, or infectious causes for aforementioned neurological symptoms were excluded by negative family history or prior history of febrile convulsion and epilepsy, cranial computed tomography (CT)/magnetic resonance imaging (MRI), electroencephalogram, lumbar puncture, biochemical, and metabolic investigations. Furthermore, all these neurological symptoms and/or signs regressed or disappeared after IVIG treatment.

Diagnoses of KD were made by two pediatricians (including at least one KD specialist) in accordance with the 2004 American Heart Association Recommendations for KD [20]. Kawasaki disease shock syndrome (KDSS) was defined as patients with KD presenting systolic hypotension for age, a sustained decrease in systolic blood pressure from baseline of ≥20%, or clinical signs of poor perfusion [24]. In order to examine whether neurological involvement contributes to the risk of IVIG resistance and CALs among patients with KD, the profiles of patients who exhibited any neurological symptoms (group A) were compared to those of gender- and admission date-matched patients without neurological involvement (group B). All available clinical details, laboratory examination parameters, echocardiogram data, treatment results, and follow-up data were systematically collected and analyzed.

In accordance with 2004 American Heart Association Recommendations, the standard IVIG therapy refers to a dose of 2 g/kg given as a single intravenous infusion after the diagnosis of KD established. Other usage of IVIG such as 1 g/kg for 2 days or 500 mg/kg for 4 days were defined as non-standard IVIG therapy. High dose of aspirin (30-50 mg/kg/day) was also given orally. After the patient’s fever had resolved, the dose of aspirin was decreased to 3–5 mg/kg/day and continued for 6–8 weeks. In patients with CALs, aspirin treatment continued until the patient exhibited no signs of coronary changes. Anticoagulants and antiplatelet therapy were also performed in accordance with 2004 American Heart Association Recommendations. Patients with recurrent or persistent fever for ≥36 h after the first IVIG administration were treated with a second course of IVIG (2 g/kg). Furthermore, high-dose pulsed intravenous methylprednisolone (20–30 mg/kg) treatment was administered for 3 days in patients with recurrent or persistent fever even after an additional IVIG. IVIG resistance was defined as persistent or recurrent fever (oral temperature ≥ 38.0 °C) or other clinical signs of KD at least 36 h but not longer than 7 days after the first IVIG infusion [25].

CALs were classified based on normalized Z scores for body surface area (BSA; units of standard deviation from the mean, normalized for BSA), as follows: no involvement (Z score < 2.0), dilation (Z score ≥ 2.0 to < 2.5), aneurysm (Z score ≥ 2.5; Z score ≥ 10 for giant aneurysm). We evaluated the maximal internal diameters of the right coronary artery (RCA), left anterior descending artery (LAD), and left circumflex coronary artery (LCX) [1]. In accordance with our institutional protocol, patients underwent standardized echocardiography by two pediatric ultrasonologists during the acute phase and 6 to 8 weeks later during follow-up evaluations in the cardiology clinic, until CALs had resolved.

### Statistical analysis

All data were analyzed using SPSS version 21.0 (SPSS Inc. Chicago, IL, USA). Quantitative data are presented as the mean and range or mean ± standard deviation (SD), while qualitative data are expressed as n/%. Shapiro–Wilk and homogeneity of variance tests were used to confirm that quantitative data from different groups exhibited a normal distribution with homogeneity of variance. Differences in quantitative data between group A and group B were assessed using independent Samples t-tests or Mann–Whitney U-tests. To assess nonparametric dichotomous and non-dichotomous variables, we used contingency tables/Fisher’s exact tests and regression analyses with dummy variables, respectively. Multivariate logistic regression analyses were performed to determine whether neurological involvement was significantly associated with IVIG resistance. These analyses included variables that significantly differed between patients with and without IVIG resistance. The level of statistical significance was set at *P* < 0.05 (two-tailed).

## Results

Neurological involvement was observed in 80 patients with KD (46 males, 34 females; M:F ratio of 1.35:1). Age in patients with neurological involvement ranged from 1 to 115 months. Sixty-percent (48/80) of patients were admitted via the Cardiology Department, 20.0% (16/80) via the Neurology Department, 16.2% (13/80) via the Pediatric Intensive Care Unit (ICU), 2.5% (2/80) via the Infectious Disease Department, and 1.3% (1/80) via other department. Neurological symptoms included headache (13/80, 16.3%), convulsions (14/80, 17.5%), somnolence (40/80, 50.1%), extreme irritability (21/80, 26.3%), signs of meningeal irritation (15/80, 18.8%), bulging fontanelles (7/80, 8.8%), and facial nerve palsy (1/80, 1.3%). The presence of hemiplegia, and sensorineural hearing loss were not observed in our cohort. Neurological involvement was present in 41.3% (33/80) of patients prior to the occurrence of familiar diagnostic features of KD, while 28.8% (23/80) presented after. Neurological symptoms represented the initial and/or predominant manifestation in 47.5% (38/80) of patients. Thirty-seven patients with neurological involvement underwent CSF examination, 12 of whom were considered to have aseptic meningitis (12/80, 15.0%) based on nucleated cell counts > 15 × 10^6^/L (mean: 110 × 10^6^/L; range: 17 to 340 × 10^6^/L). Among these 12 patients, 66.7% (8/12) exhibited lymphocyte predominance (mean: 53.9%, range: 6.0 ~ 100.0%), while the remaining 33.3% (4/12) exhibited neutrophil predominance (mean: 33.8%, range: 0.0 ~ 93%). The remaining 25 patients had normal CSF results. Protein, glucose, chloride, and lactate dehydrogenase (LDH) levels in the CSF were within the normal range. The results of bacteriologic and viral investigations in the CSF were negative in all patients. Biochemical and metabolic investigations (e.g., blood glucose, electrolytes, ammonia, amino acid chromatography, organic acids) yielded normal results. Almost all KD patient exhibiting neurological involvement had received cranial CT examinations but no positive findings were noted. Meningeal enhancement was also not observed in any patients with aseptic meningitis. Meanwhile, 14 patients suffering from convulsions had further underwent MRI, but no abnormalities were found. In patients with CSF examination, the proportion of males was significantly higher among patients with KD with abnormal results of CSF than without; clinical characteristics and laboratory profiles showed no differences, as summarized in supplemental material 1.

A detailed comparison of clinical data between groups A and B is presented in Table 1. There were noted no significant differences in the gender ratio, average age, fever duration on admission/before initial IVIG, incidence of incomplete KD or typical clinical features, and the rate of delayed IVIG treatment between the groups (all *P* > 0.05). The occurrence of KDSS was significantly higher in patients from group A than that those from group B (12.5% vs 0.2%, *P* < 0.001). The duration of antibiotic treatment was significantly longer in group A than in group B (6.4 ± 2.5 vs. 4.8 ± 2.2 days, P < 0.001). In addition, rates of IVIG resistance (27.5% vs. 13.7%, *P* = 0.003) and intravenous steroid treatment (16.3% vs. 7.4%, *P* = 0.016) were higher in group A than in group B. Similar results were obtained when comparing patients in the two subgroups admitted via Non-Cardiology departments (16.3% vs. 1.6%, P<0.001). Prior to the initial IVIG treatment, the following were significantly higher in group A than in group B: white blood cell (WBC), neutrophil percentages, C-reactive protein (CRP), total bilirubin (TB), and creatinine (Cr) (all *P*<0.005). However, hemoglobin (Hb) levels, albumin (ALB) levels, and lymphocyte percentage were lower in group A than in group B (all P<0.005). Rates of cardiac complications including CALs, valve regurgitation, cardiac enlargement, and pericardial effusion were relatively higher in group A than in group B. These differences were not statistically significant. The *z* scores of CALs in patients from group A also did not significantly differ from that in patients from group B (1.59 ± 0.40 vs 1.57 ± 0.37, *P* = 0.709). In comparison with patients of group B, somnolence as one subtype presentation in KD with neurological involvement showed a higher incidence of IVIG resistance but similar incidence of CALs, no significant difference of IVIG resistance and CALs was observed in patients for other subtype presentations (Table 2). Additionally, it found that neurological involvement was not an independent risk factor for IVIG resistance in patients with KD (Supplementary material 2 and 3).

**Table 1:** The comparison of demographic, clinical characteristic and laboratory results of Kawasaki disease between the two groups

	Group A^a^	Group B^b^	*P* value
**Clinical characteristic**			
Patients, n	80	512	–
Sex, male, n(%)	46(57.5)	296(57.8)	0.526
Age, months	33.0 ± 26.8	30.4 ± 23.4	0.437
Fever duration on admission, days	5.5 ± 3.0	5.5 ± 2.8	0.893
Incomplete Kawasaki disease, n(%)	34(42.5)	192(37.5)	0.390
Kawasaki disease shock syndrome, n(%)	10(12.5)	1(0.2)	<0.001^*^
**Typical clinical manifestations**			
Fever, n(%)	80(100.0)	512(100.0)	–
Rash, n(%)	60(75.0)	395(77.1)	0.670
Edema & erythema of the extremities, n(%)	46(57.5)	297(58.0)	0.863
Bilateral bulbar conjunctival injection, n(%)	70(87.5)	464(90.6)	0.480
Erythema of oral and pharyngeal mucosa, n(%)	72(90.0)	470(91.8)	0.524
Cervical lymphadenopathy, n(%)	38(47.5)	220(43.1)	0.470
**Treatment**			
The duration of antibiotic, days	6.4 ± 2.5	4.8 ± 2.2	<0.001^*^
Fever duration before IVIG, days	6.4 ± 4.0	6.3 ± 3.0	0.782
The delayed treatment of initial IVIG (>10 days)	4(5.0)	21(4.1)	0.762
Failure to respond to initial IVIG therapy, n(%)	22(27.5)	70(13.7)	0.003^*^
Patients from Cardiology Department, n(%)	9 (11.3)	62(12.1)	0.837
Patients from non-Cardiology Department, n(%)	13 (16.3)	8(1.6)	<0.001^*^
Intravenous and/or Oral steroid treatment, n(%)	13(16.3)	38(7.4)	0.016^*^
**Cardiac complications**			
Coronary artery lesions, n(%)	12(15.0)	50(9.8)	0.220
Dilatation, n(%)	11(13.8)	45(8.8)	0.180
Aneurysms, n(%)	1(1.3)	5(1.0)	0.821
Valve regurgitation, n(%)	8(10.0)	32(6.3)	0.229
Cardiac enlargement, n(%)	10(12.5)	37(7.2)	0.118
Pericardial effusion, n(%)	4(5.0)	12(2.3)	0.253
**Blood examination features**			
White blood cell count, × 10^9^/L	15.5 ± 5.8	14.3 ± 5.4	0.042^*^
Neutrophil rate, %	70.3 ± 17.0	66.1 ± 14.7	0.014^*^
Lymphocyte rate, %	19.6 ± 12.3	25.1 ± 12.9	<0.001^*^
C-reactive proteins, mg/L	96.0 ± 53.1	79.3 ± 47.8	0.003^*^
Erythrocyte sediment rate, mm/h	69.4 ± 34.1	65.1 ± 29.1	0.326
Platelet, ×10^9^/L	325.0 ± 117.0	343.9 ± 115.0	0.164
Hemoglobin, g/L	104.0 ± 13.3	108.4 ± 10.9	0.001^*^
Alanine transaminase, U/L	75.2 ± 83.9	67.8 ± 84.4	0.516
Aspartate transaminase, U/L	58.9 ± 88.3	49.5 ± 57.0	0.233
Albumin, g/L	34.4 ± 7.0	37.7 ± 5.0	<0.001^*^
Total bilirubin, umol/L	10.3 ± 15.3	7.7 ± 7.9	0.026^*^
Cr, umol/L	33.5 ± 14.6	28.2 ± 13.8	0.003^*^
Serum sodium, mmol/L	135.3 ± 4.8	136.3 ± 7.1	0.098

The data are presented as mean ± standard deviation (SD) for quantitative variables and as n/% for qualitative data as appropriate. ^*^P < 0.05

*IVIG* Intravenous immunoglobulin

^a^Group A, KD patients had neurological involvement

^b^Group B, KD patients had no neurological involvement

**Table 2:** Comparison of IVIG resistance and CALs of KD between patients with and without neurological involvement

	Neurological involvement (*n* = 80)							Group B (*n* = 512)
	Somnolence (*n* = 40, 50.1%)	Convulsion (*n* = 14,17.5%)	Headache (*n* = 13, 16.3%)	Extreme irritability (*n* = 21, 26.3%)	Positive sign of meningeal irritation (*n* = 15, 18.8%)	Bulgin fontanel (*n* = 7, 8.8%)	Facial nerve palsy (*n* = 1,1.3%)	
IVIG resistance, n(%)	14(35.0) ^a^	3(21.4) ^b^	4(30.8) ^b^	6(28.6) ^b^	4(26.7) ^b^	0(0.00) ^b^	0(0.00) ^b^	70(13.9)
CALs, n(%)	4(10.0) ^b^	2(14.3) ^b^	0(0.00) ^b^	3(14.3) ^b^	4(26.7) ^b^	1(14.3) ^b^	0(0.00) ^b^	50(9.6)

*IVIG* Intravenous immunoglobulin, *CALs* Coronary artery lesions

^a^There was a statistic significant difference between neurological involvement and group B

^b^There was no statistic significant difference between neurological involvement and group B

## Discussion

To our knowledge, the present study is the first to systemically analyze neurological involvement among patients with KD, and whether such involvement is associated with an increased risk of IVIG resistance and CALs. Our findings indicate that the incidence of neurological involvement was relatively low among patients with KD (5.1%). In many of these patients, neurological involvement was associated with diffuse clinical symptoms and accounted for the initial and/or predominant presentation. Although we observed no significant delays in diagnosis or IVIG treatment between those with and without neurological involvement, patients with neurological involvement (particularly those admitted via non-cardiology departments) exhibited a higher incidence of non-standard IVIG therapy and IVIG resistance. Inconsistent with our hypothesis, we observed no significant differences in the rate of CALs occurrence between patients with and without neurological involvement.

The neurological manifestations of KD were diffuse, presenting as headache, convulsions, somnolence, extreme irritability, signs of meningeal irritation, bulging fontanelles, and facial nerve palsy. The presence of hemiplegia and sensorineural hearing loss were not found in our cohort. Fortunately, almost all above neurological symptoms regressed or disappeared after IVIG treatment and the overall prognosis of patients with KD exhibiting neurological involvement was satisfactory. Although the incidence of incomplete KD was higher than expected (42.5%) in patients with neurological involvement, IVIG treatment was delayed in only 5.0% of patients. Our findings indicated that levels of inflammatory markers (WBC count, neutrophil percentage, CRP levels, etc.) were significantly increased in patients with neurological involvement. In addition, 15.0% of patients with neurological involvement undergoing CSF examination were diagnosed with aseptic meningitis based on increases in nucleated cell counts without alterations in other CSF indices. A total of 66.7% exhibited lymphocyte predominance, while the remaining patients exhibited neutrophil predominance. In patients undergoing CSF examination, those with aseptic meningitis only were more likely females, whereas their typical clinical manifestations of KD, laboratory results, treatment, and prognosis did not differ from those without. The prevalence of delayed treatment in patients with neurological involvement was similar to patients without neurological involvement, but a second round of IVIG and/or steroid treatment were more often observed in patients with neurological involvement. However, in subtype neurological symptoms, patients only presenting somnolence were more likely to develop a higher incidence of IVIG resistance and patients with other neurological symptoms showed no significant difference, inconsistent with prior studies [5, 14]. In addition, almost all KD patient exhibiting neurological involvement had received cranial CT examinations but no positive findings were noted. Meningeal enhancement was also not observed in any patients with aseptic meningitis. Meanwhile, 14 patients suffering from convulsions had further underwent MRI, but no abnormalities were found. However, despite the rarity, some severe and fatal cerebral vascular complications such as artery aneurysms and/or arterial stroke had been reported in KD patients [26]. Therefore, clinicians should also alert the possibility of aforementioned severe neurological complications, particularly in KD patients with prolonged fever or severe progression, and some specific neuroimaging evaluation such as magnetic resonance angiography (MRA), angiography and even single-photon emission computed tomography (SPECT), should be performed if it is necessary.

As expected, our data indicated that KD patients with neurological involvement appear to have a higher incidence of IVIG resistance. Despite the underlying mechanisms not entirely clear, several explanations could be hypothesized. Firstly, consistent with the study conducted by Korematsu et al. [27], increased levels of inflammatory markers and nucleated cell counts in the CSF were observed in KD patients with neurological involvement. Indeed, increase in the inflammatory burden has been found to be associated with IVIG resistance. Secondly, despite the incidence of delay in IVIG administration being relatively lower, a high incidence of non-standard IVIG therapy was observed, particularly in patients admitted via the non-Cardiology departments. It was noteworthy that 100% patients with neurological involvement admitted via the Neurology Department received non-standard IVIG treatment (1 g/kg for 2 days) due to suspected immune disease, while more than 50% of patients admitted via the Pediatric ICU and Infectious Disease Department received IVIG treatment at a dose of 500 mg/kg for an initial consideration of severe infectious disease. Substantial evidence has suggested that non-standard treatment could predispose KD patients to a higher risk of IVIG resistance [28–31]. However, a further multivariate logistic regression analysis failed to demonstrate neurological involvement as an independent risk factor for IVIG resistance in patients with KD. This controversial result may be explained by a correlation between serum inflammatory markers and the presence of neurological involvement. Alternatively, neurological involvement may be less sensitive as a biomarker for IVIG resistance prediction compared to serum inflammatory markers such as WBC count, neutrophil percentages, CRP level, TB level, and Cr level.

Another important finding in our study was the negative associations of neurological involvement including the subtype of neurological presentations and CALs, which appears to be inconsistent with previous studies [5, 14]. However, almost all these studies represented sporadic cases of KD with neurological involvement and did not provide statistical support for the relationship between CALs and neurological involvement [7–10]. Stowe reported that the rate of CALs was more than twice as high in patients with KD presenting with facial nerve palsy than in those with untreated KD [14]; however, the study might have been affected by selection bias. In contrast, patients with KD presented diverse neurological manifestations in the present study, more accurately reflecting the outcome of KD with neurological involvement. It was demonstrated that IVIG treatment could reduce the occurrence of coronary artery abnormalities in the acute phase of KD [32]. Even non-standard IVIG treatment (500 mg/kg for 4 days, or 1 g/kg for 2 days) also tends to reduce the incidence of new coronary artery abnormalities [28]. Therefore, the negative associations between neurological involvement and CALs in KD patients in this study could be partly explained by the timely IVIG treatment despite a high incidence of non-standard IVIG therapy observed.

This study possesses some limitations of note, including its retrospective and cross-sectional design. In addition, given the prevalence of neurological involvement in our patients, our study might have been affected by selection bias. Indeed, as our institution is the largest pediatric medical center in Southwest China, we encounter a greater proportion of complex KD cases than other institutions. Lastly, the definitions of some neurological symptoms including somnolence, extreme irritability and headache were to be with a certain degree of subjectivity, which may not be completely avoided in the present study. However, lots of efforts had been made to avoid this limitation. Several scales and/or tools were applied to reduce the possibilities of subjective evaluations. Moreover, the proportion of patients with KD exhibiting neurological involvement affected by subjective judgement was relatively small, and it may not affect our main findings and the conclusions of our study.

## Conclusion

Our findings indicate that the incidence of neurological involvement is relatively low among patients with KD. Although no significant delays in diagnosis/IVIG treatment were observed, patients with neurological involvement were more likely to develop IVIG resistance and exhibit more severe inflammatory burden than patients without neurological involvement. The overall prognosis of patients with KD exhibiting neurological involvement was satisfactory, as we observed no associations between neurological involvement and the development of CALs. Owing to atypical clinical profile of some KD patients in early disease stage and limited knowledge of clinicians on the neurological involvements in KD, the usage of non-standard IVIG was relatively common in this cohort due to a primary misdiagnosis, particularly among patients from non-Cardiology departments. Therefore, pediatricians should improve the recognition and understanding of the diverse clinical manifestations of KD patients with neurological involvements, which may contribute to the prompt usage of standard IVIG therapy and in turn decrease the incidence of IVIG resistance in this populations.

## Supplementary information

**Additional file 1: Supplemental material 1.** The comparison of clinical characteristic and laboratory results of patients with Kawasaki disease underwent CSF examination.

**Additional file 2: Supplemental material 2.** Comparison of clinical data between the groups of IVIG-response and IVIG-resistance in KD.

**Additional file 3: Supplemental material 3.** A multivariate logistic regression model for IVIG resistance in patients with KD.

## Data Availability

All data generated or analyzed during this study are included in this published article and the supplementary files.

## Abbreviations

KD: Kawasaki disease
KDSS: Kawasaki disease shock syndrome
CALs: Coronary artery lesions
IVIG: Intravenous immunoglobulin
WCSUH-SCU: West China Second University Hospital of Sichuan University
BSA: Body surface area
CSF: Cerebrospinal fluid
FLACC scale: Face, Legs, Activity, Cry, Consolability scale
RCA: Right coronary artery
LAD: Left anterior descending artery
LCX: Left circumflex coronary artery
LDH: Lactate dehydrogenase
WBC: White blood cell
CRP: C-reactive protein
Hb: Hemoglobin
Cr: Creatinine
TB: Total bilirubin
ALB: Albumin
MRI: Magnetic resonance imaging
MRA: Magnetic resonance angiography
SPECT: Single-photon emission computed tomography
