# Supplementary material for: Neurological involvement in Kawasaki disease: a retrospective study
Source: Pediatr Rheumatol Online J. 2020 Jul 14;18:61. doi: 10.1186/s12969-020-00452-7 (PMC7362431; doi:10.1186/s12969-020-00452-7)
Supplement: Supplementary file 1 — Additional file 1: Supplemental material 1. The comparison of clinical characteristic and laboratory results of patients with Kawasaki disease underwent CSF examination. [file 12969_2020_452_MOESM1_ESM.docx]

**Table** **1** The comparison of clinical characteristic and laboratory results of patients with Kawasaki disease underwent CSF examination.

| **Clinical characteristic** | Patients with normal CSF | Patients with abnormal CSF | P value |
| --- | --- | --- | --- |
| Patients, n | 25 | 12 | - |
| Sex, male, n(%) | 18(72.0) | 4(33.3) | 0.036 |
| Age, months, n(%) | 28.8±30.6 | 34.0±31.9 | 0.639 |
| Fever duration on admission, days | 5.7±3.5 | 5.6±3.6 | 0.939 |
| Incomplete Kawasaki disease, n(%) | 10(40.0) | 7(58.3) | 0.482 |
| **Typical clinical manifestations** |  |  |  |
| Fever, n(%) | 25(100.0) | 12(100.0) | - |
| Rash, n(%) | 19(76.0) | 10(83.3) | 1.000 |
| Edema & erythema of the extremities, n(%) | 10(40.0) | 5(41.7) | 1.000 |
| Bilateral bulbar conjunctival injection, n(%) | 21(84.0) | 7(58.3) | 0.116 |
| Erythema of oral and pharyngeal mucosa, n(%) | 22(88.0) | 9(75.0) | 0.367 |
| Cervical lymphadenopathy, n(%) | 16(64.0) | 6(50.0) | 0.488 |
| **Treatment** |  |  |  |
| Fever duration before IVIG, mean±SD, days | 6.4±4.0 | 6.3±3.0 | 0.782 |
| The delayed initial IVIG (＞10 days) | 1(4.2) | 1(8.3) | 1.000 |
| Failure to respond to initial IVIG therapy, n(%) | 8(32.0) | 5(41.7) | 0.716 |
| **Coronary artery lesions, n(%)** | 4(16.0) | 1(8.3) | 1.000 |
| **Blood examination features** |  |  |  |
| White blood cell count, ×10^9^/L | 16.3±6.7 | 16.0±5.5 | 0.897 |
| Neutrophil rate, % | 70.8±17.1 | 68.8±21.0 | 0.761 |
| Lymphocyte rate, % | 19.3±14.3 | 15.1±11.3 | 0.378 |
| C-reactive proteins, mg/L | 103.3±48.9 | 88.4±57.2 | 0.427 |
| Erythrocyte sediment rate, mm/h | 60.6±37.3 | 72.6±37.8 | 0.384 |
| Platelet, ×10^9^/L | 314.0±84.4 | 326.1±127.7 | 0.734 |
| Hemoglobin, g/L | 101.0±13.1 | 104.1±13.4 | 0.511 |
| Alanine transaminase, U/L | 63.1±48.5 | 33.5±18.5 | 0.061 |
| Aspartate transaminase, U/L | 61.0±58.3 | 36.1±15.7 | 0.177 |
| Albumin, g/L | 33.4±5.3 | 31.3±12.2 | 0.491 |
| Total bilirubin, umol/L | 7.9±12.8 | 10.4±16.0 | 0.631 |
| Cr, umol/L | 31.5±10.3 | 32.3±10.5 | 0.825 |
| Serum sodium, mean±SD, mmol/L | 135.6±2.8 | 136.1±5.5 | 0.728 |

IVIG: intravenous immunoglobulin. CSF: cerebrospinal fluid.

The data are presented as mean ± standard deviation (SD) for quantitative variables and as n/% for qualitative data as appropriate.
