# Supplementary material for: Neurological involvement in Kawasaki disease: a retrospective study
Source: Pediatr Rheumatol Online J. 2020 Jul 14;18:61. doi: 10.1186/s12969-020-00452-7 (PMC7362431; doi:10.1186/s12969-020-00452-7)
Supplement: Supplementary file 2 — Additional file 2: Supplemental material 2. Comparison of clinical data between the groups of IVIG-response and IVIG-resistance in KD. [file 12969_2020_452_MOESM2_ESM.docx]

**Supplemental material 1.** Comparison of clinical data between the groups of IVIG-response and IVIG-resistance in KD

|  | **IVIG-responsive(n=500)** | **IVIG-resistance(n=92)** | ***P* value** |
| --- | --- | --- | --- |
| Male, n(%) | 290(58.0) | 52(56.5) | 0.819 |
| Age, years | 29.5±22.9 | 37.5±27.7 | 0.050 |
| **Clinical manifestations** |  |  |  |
| Rash, n(%) | 380(76.0) | 75(81.5) | 0.283 |
| Edema & erythema of the extremities, n(%) | 294(58.8) | 48(52.2) | 0.087 |
| Bilateral bulbar conjunctive injection, n(%) | 449(89.8) | 79(85.9) | 0.274 |
| Erythema of oral and pharyngeal mucosa, n(%) | 454(90.8) | 88(95.7) | 0.154 |
| Cervical lymphadenopathy, n(%) | 209(42.0) | 49(53.3) | 0.052 |
| Fever duration before IVIG administration, days | 6.4±3.1 | 5.9±2.2 | 0.062 |
| Neurological involvement, n(%) | 58(11.6) | 22(23.9) | 0.003^*^ |
| Incomplete KD, n(%) | 195(39.0) | 31(33.7) | 0.353 |
| Coronary artery lesions, n(%) | 48(9.6) | 13(14.1) | 0.193 |
| **The laboratory results before initial IVIG** |  |  |  |
| White blood cell(WBC),×10^9^/L | 14.4±5.3 | 15.1±6.1 | 0.251 |
| Neutrophil count, % | 65.3±14.8 | 74.3±14.7 | ＜0.001^*^ |
| Lymphocyte count, % | 25.7±12.8 | 16.7±11.1 | ＜0.001^*^ |
| Hematocrit ratio, % | 31.8±3.3 | 31.4±3.7 | 0.282 |
| Hemoglobin, g/L | 107.9±11.1 | 107.1±12.3 | 0.566 |
| Platelet count, ×10^9^/L | 349.6±115.4 | 294.9±104.1 | ＜0.001^*^ |
| C-reactive protein, mg/L | 78.7±48.0 | 97.4±51.1 | 0.001^*^ |
| Erythrocyte sedimentation rate, mm/h | 65.4±29.0 | 67.2±34.4 | 0.621 |
| Aspartate aminotransferase, U/L | 50.0±64.0 | 54.9±49.9 | 0.493 |
| Alanine aminotransferase, U/L | 66.4±84.4 | 82.0±83.5 | 0.109 |
| Albumin, g/L | 37.9±4.9 | 34.1±6.6 | ＜0.001^*^ |
| Total bilirubin, mmol/L | 7.1±7.3 | 13.1±15.5 | ＜0.001^*^ |
| Cr, mmol/L | 28.3±14.3 | 32.3±11.3 | 0.016^*^ |
| Serum sodium, mmol/L | 136.4±7.3 | 134.8±3.8 | 0.039^*^ |

IVIG: Intravenous immunoglobulin; KD: Kawasaki disease. The data are presented as mean ± standard deviation (SD) for quantitative variables and as n/% for qualitative data as appropriate. ^*^*P*<0.05.
