# Supplementary material for: Neurological involvement in Kawasaki disease: a retrospective study
Source: Pediatr Rheumatol Online J. 2020 Jul 14;18:61. doi: 10.1186/s12969-020-00452-7 (PMC7362431; doi:10.1186/s12969-020-00452-7)
Supplement: Supplementary file 3 — Additional file 3: Supplemental material 3. A multivariate logistic regression model for IVIG resistance in patients with KD. [file 12969_2020_452_MOESM3_ESM.docx]

**Supplemental material 2.** A multivariate logistic regression model for IVIG resistance in patients with KD

|  | **β** | **SE** | **Wals** | **P value** | **OR** | **95% CI** |
| --- | --- | --- | --- | --- | --- | --- |
| Neurological involvement | 0.008 | 0.083 | 0.008 | 0.928 | 1.008 | 0.856-1.186 |
| Neutrophil percentage | -0.047 | 0.013 | 13.812 | ＜0.001 | 0.954 | 0.930-0.978 |
| Platelet | 0.003 | 0.001 | 5.889 | 0.015 | 1.003 | 1.001-1.006 |
| C-reaction protein | 0.003 | 0.003 | 1.148 | 0.284 | 1.003 | 0.997-1.009 |
| Total Bilirubin | -0.032 | 0.012 | 6.658 | 0.010 | 0.968 | 0.945-0.992 |
| Albumin | 0.087 | 0.026 | 11.176 | 0.001 | 1.091 | 1.037-1.147 |
| Creatinine | 0.003 | 0.009 | 0.146 | 0.703 | 1.004 | 0.986-1.032 |
| Serum sodium | 0.000 | 0.016 | 0.000 | 0.999 | 1.000 | 0.969-1.032 |

IVIG: Intravenous immunoglobulin; KD: Kawasaki disease.
